# Radiation induced angiosarcoma a sequela of radiotherapy for breast cancer following conservative surgery

**DOI:** 10.1186/1477-7800-3-26

**Published:** 2006-09-11

**Authors:** M Tahir, P Hendry, L Baird, NA Qureshi, D Ritchie, P Whitford

**Affiliations:** 1 Department of Surgery, Crosshouse Hospital, Kilmarnock, UK

## Abstract

Radiation induced angiosarcomas (RIA) can affect breast cancer patients who had radiotherapy following conservative breast surgery. They are very rare tumors and often their diagnosis is delayed due to their benign appearance and difficulty in differentiation from radiation induced skin changes. Therefore it is very important that clinicians are aware of their existence. We report here a case of RIA followed by discussion and review of literature.

## Background

Angiosarcomas are rare tumors of endovascular origin. Primary angiosarcomas account for only 0.04% of breast tumors. It affects a younger age group of 20–40 yrs. Secondary angiosarcoma can be induced by radiotherapy. Cases of radiation induced angiosarcoma (RIA) have been reported in women who had radiotherapy for breast cancer. RIA occurs in an older age group, with a mean age of 68 years [1]. It is difficult to diagnose due to its rarity, benign appearance and difficulty in differentiation from radiation induced changes in the skin [2].

We report a case of Angiosarcoma in a 78 years old woman. She had radiotherapy for breast cancer following conservative surgery and axillary node clearance.

## Case report

### Clinical history

In April 1996 this 78-year-old woman was diagnosed with a poorly differentiated lobular carcinoma in the upper outer part of the left breast. Clinically it measured 15 mm. She was treated with conservative breast surgery and level III axillary node clearance. Tumor margins were clear and all lymph nodes were negative. Tamoxifen was commenced at 20 mg OD.

A course of postoperative radiotherapy was arranged due to breast conservation surgery. She received 46 Gy in 23 fractions over 35 days using paired glancing megavoltage fields. This treatment was completed in July 1996 and was followed by a boost to the site of excision of a further 12 Gy in 4 fractions over 7 days using 9MEV electrons. Chemotherapy was discussed but the patient did not want to pursue this option.

**Figure 1:**
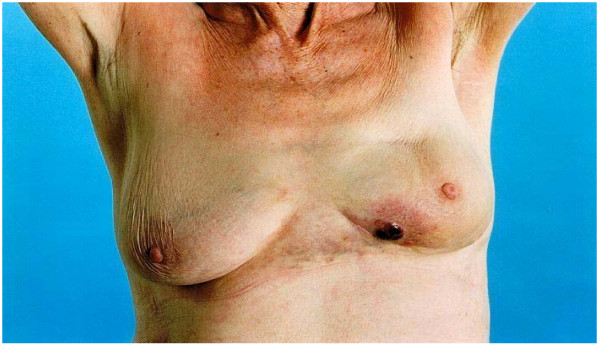
Radiation induced Angiosarcoma (left breast) following conservative surgery for breast cancer in a 78 years old lady.

She remained in regular follow up with no evidence of local or regional recurrence. In June 2005 during a routine follow up appointment she reported a 2 cm area of purple skin lesion at the lower inner quadrant of the left breast (figure 1), with a history of it being there for about 3 months. It was associated with peau d'orange and soft tissue thickening extending from the lower inner quadrant across the midline. Mammography was not performed, as she was too tender. Ultrasound showed no evidence of a lesion deep to the abnormality. Freehand core biopsies were taken of the area. Pathology demonstrated the presence of malignancy in keeping with high-grade angiosarcoma.

Staging investigations including CT chest and abdomen, CXR and bone scan none showed any evidence of metastasis. She had wide surgical excision and reconstruction with a Vertical Rectus Abdominus Myocutaneous flap (VRAM flap). She has completed her first year follow up without any recurrence.

### Pathologic findings

**Figure 2:**
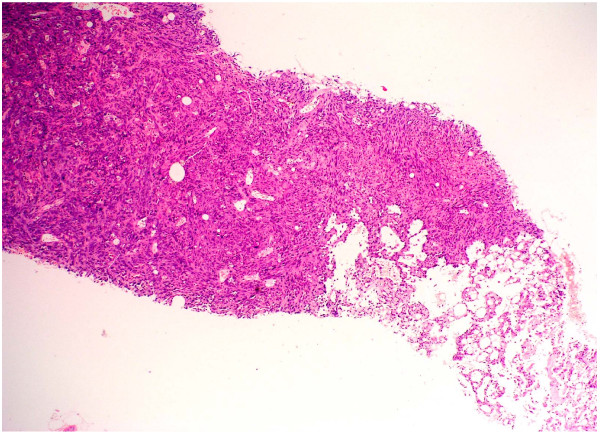
Histological sections from the biopsy at low power (4 × magnifications) show infiltration by a tumor with both solid areas and neoplastic vascular channels.

**Figure 3:**
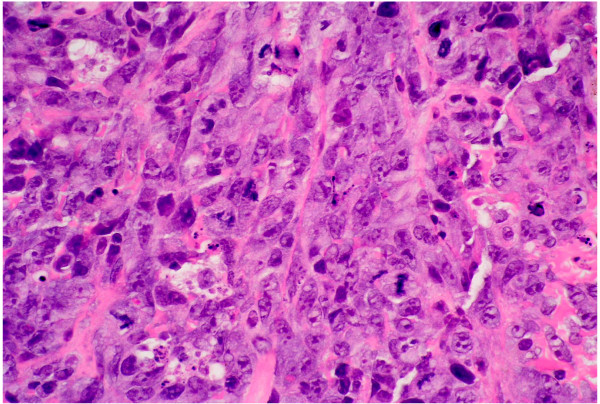
At high power magnification (×40) there are highly pleomorphic cells with prominent nucleoli. Many mitotic figures are seen (arrows). Some individual apoptotic cells are also present.

**Figure 4:**
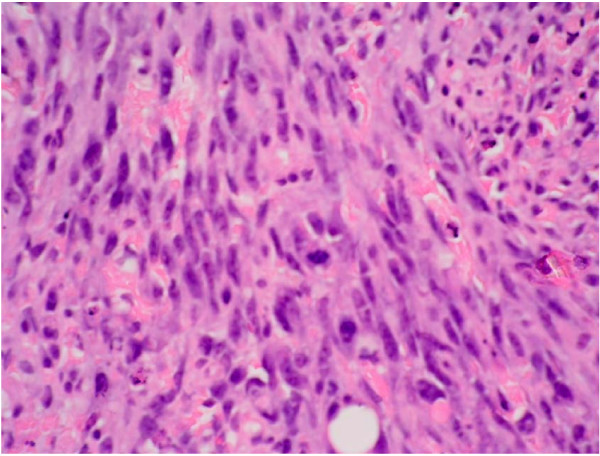
This shows a high power view (×40 magnification) of an area with spindle cell morphology.

**Figure 5:**
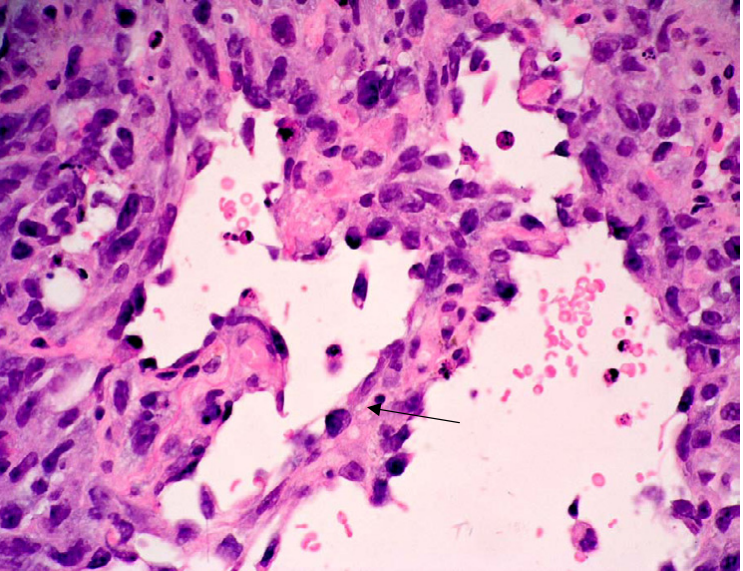
This high power view (×40 magnification) shows neoplastic vascular channels. These irregular channels interconnect and are lined by a single layer of highly atypical endothelial cells (see arrow).

Histological examination following routine processing and staining with haemotoxylin and eosin shows malignant tumor (figure 2). Although this tumor is predominantly solid, comprising highly pleomorphic cells (figure 3) and areas with spindle cell morphology (figure 4) neoplastic vascular channels are also identified (figure 5). Immunohistochemical staining for CD31, CD34 and factor viii confirm endothelial differentiation both within the solid tumor component and the recognizable vascular channels. The possible differential diagnoses of carcinoma and malignant melanoma are excluded by the negativity of other stains (MNF and S100 respectively). The histological features are those of high grade angiosarcoma.

**Figure 6:**
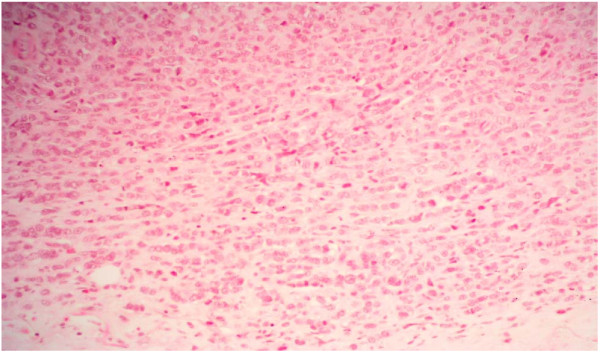
At medium power (×20 magnification), histology from the original breast excision biopsy shows infiltrating strands of tumor cells with some pleomorphism. The tumor also forms circumscribed nodules.

Histology from the original breast excision biopsy (figure 6) shows a lobular carcinoma, with strong oestrogen receptor positivity. This shows no similarity to the newly arising angiosarcoma.

## Discussion and review of literature

Radiation induced sarcomas were first reported in literature in 1929[3] and the first case of sarcoma after radiotherapy for breast cancer was reported by Warren & Sommer in 1936[4]. Cahan et al [5] described the diagnostic criteria for Radiation induced sarcomas which include a previous history of radiotherapy with a latency period of several years (5 or more), development of sarcoma within a previous irradiated field and a histologic confirmation. This criterion was modified by Arlen et al [6] to include tissues adjacent to the radiated field and a shorter latency period of 3–4 years. Radiation induced angiosarcomas are characterized by their aggressive nature and most are high grade tumors [7,8]. They can develop after many years in women who have previously had radiotherapy for breast cancer.

The etiology of radiation induced angiosarcomas in the breast remains controversial. Some investigators believe that many of the reported cases are actually metaplastic variants of the original Cancer [9,10]. Radiation induced metaplastic transformation is thought to be related to irreversible DNA damage [11]. Immunohistochemical stains of tumor tissue for factor VIII – related antigen & Ulex lectin stains are required to confirm the diagnosis of angiosarcoma and to differentiate it from the metaplastic recurrent breast cancer [7,8]. In our case we have performed these tests to confirm the diagnosis.

Most cases of RIA occur in association with a total radiation dose in the range of 40–50 Gy [8,12]. It is difficult to analyze the relationship between the total radiation dose, individual fraction dose and the incidence of RIA, due to the rarity of cases in the literature and the difficulty in retrieving information many years after the primary treatment. A minimum total dose of 10 Gy in conventional doses per fraction appears necessary to result in RIA [13,14].

The incidence of RIA following radiotherapy to the breast varies from 0.05 to 0.2 % [15-17] with an average latency period reported as 12.5 years [18,19].

The presentation of RIA is often a cutaneous or subcutaneous lesion, painless, flat or nodular, bluish or purplish similar to benign angiomas, small hematomas or atypical telangiectasis [20,21]. Its diagnosis is often delayed by 8–12 months [8,11] due to a lack of specific symptoms and low suspicion following a long latency period after diagnosis of original tumor. Additional reasons cited for delayed diagnosis include difficulty in detecting the tumor in previously irradiated tissue and inadequate biopsies [2].

A high index of suspicion, careful patient evaluation and adequate biopsy tissue for pathologic diagnosis is mandatory. Mammography is typically negative in these cases [2] and diagnosis can be confirmed only by histopathological study of the biopsy. Cytology is generally misleading and not very informative [2].

There is general consensus in literature that the only logical treatment of RIA regardless of histologic type is wide surgical resection i.e. Mastectomy usually with a latissimus dorsi flap reconstruction. Conservative treatment even with negative margins exposes the patient to early recurrences and metastatic spread [2,17-20,22].

Adjuvant Chemotherapy in RIA has so far produced disappointing results [22].

Radiation therapy has been avoided in these cases secondary to concerns about the toxicity of repeated treatment. However recently some encouraging results have been achieved with the use of hyperfractionated radiotherapy [23].

Median survival in RIA is reported from1.5 to 2.5 years [17]. Recurrence rates approach 70 % and 2-year disease free survival ranges from 0–35 % in various series [15,16].

## Conclusion

This lesion is still rare, but with the increase in conservative breast surgery and radiotherapy, the incidence of RIA is likely to increase. Due to benign looking nature of the tumor in initial stages only a high index of suspicion and generous tissue biopsy can lead to early diagnosis. From review of the literature it is evident that early diagnosis and aggressive surgical approach can lead to a better prognosis. Chemotherapy has yet achieved no role in the treatment of these tumors but recently some encouraging results are seen with the use of Hyperfractionated radiotherapy and further work needs to be done in this regards.

## Competing interests

The author(s) declare that they have no competing interests.
